# Semaphorins and Their Receptors: From Axonal Guidance to Atherosclerosis

**DOI:** 10.3389/fphys.2018.01236

**Published:** 2018-10-12

**Authors:** Shuhong Hu, Li Zhu

**Affiliations:** ^1^Cyrus Tang Hematology Center, Collaborative Innovation Center of Hematology, Soochow University, Suzhou, China; ^2^State Key Laboratory of Radiation Medicine and Protection, Soochow University, Suzhou, China

**Keywords:** Semaphorins, atherosclerosis, inflammation, cell infiltration, angiogenesis

## Abstract

Semaphorins are a large family of secreted, transmembrane, or GPI-anchored proteins initially identified as axon guidance cues signaling through their receptors, neuropilins, and plexins. Emerging evidence suggests that beyond the guidance, they also function in a broad spectrum of pathophysiological conditions, including atherosclerosis, a vascular inflammatory disease. Particular semaphorin members have been demonstrated to participate in atherosclerosis via eliciting endothelial dysfunction, leukocyte infiltration, monocyte-macrophage retention, platelet hyperreactivity, and neovascularization. In this review, we focus on the role of those semaphorin family members in the development of atherosclerosis and highlight the mechanistic relevance of semaphorins to atherogenesis.

## Introduction

Atherosclerosis is a chronic inflammatory process initiated by endothelial dysfunction and the secretion of cytokines and chemokines, and it allows the subendothelial accumulation of low-density lipoprotein (LDL) (Gibson et al., 2018). The deposited LDL is then oxidized and becomes oxidized LDL (ox-LDL) that potently stimulates cytokine release from intimal endothelial cells and facilitates leukocyte adhesion and transmigration (Singh et al., 2002; Paoletti et al., 2004). The infiltrated monocytes differentiate into macrophages that uptake oxLDL and other forms of modified LDL (Rader and Pure, 2005), subsequently forming foam cells that are the constitutes of fatty streaks (Siegel-Axel et al., 2008). Accumulating immune cells including macrophages and T cells fuel immune response and inflammation, promoting the transition from a fatty streak to a complex atherosclerotic plaque (Jonasson et al., 1986; Samson et al., 2012). The ultimate stage of atherosclerotic disease is hallmarked by plaque rupture and thrombus formation that may lead to acute fatal coronary syndrome (ACS), myocardial infarction (MI), and stroke. To date, a vast number of proteins have been shown to participate in the onset and progression of atherosclerosis, including those from plasma, blood cells, and the vascular wall, that fall into the classical class of molecules that have established current understanding of atherogenesis (Geovanini and Libby, 2018). However, additional molecules are being identified, shedding new light on the modulation and control of atherosclerosis.

**FIGURE 1:**
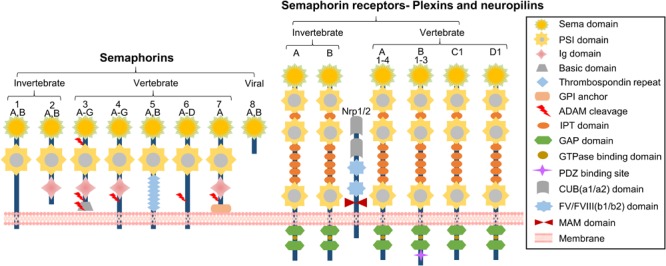
Schematic representation of semaphorins and semaphorin receptors. The semaphorin family can be divided into eight classes. Classes 1 and 2 are found in invertebrates and classes 3–7 belong to vertebrates. Classes 2 and 3, and the viral semaphorin 8 are secreted whereas classes 4–6 are transmembrane proteins. Class 7 is the only GPI-anchored protein. Each semaphorin consists of a large sema domain, a plexin-sema-integrin domain (PSI), immunoglobulin (Ig)-like domains, and thrombospondin repeats. Semaphorins signal through their receptors, plexins. Plexins A and B are found in invertebrates. Vertebrates have plexins A1–4, plexins B1–3, PlexinC1, and PlexinD1. Plexins contain a sema domain, a PSI domain, and an Ig-like, plexins, transcription factors (IPT) domain. Structurally, the cytoplasmic domain of the plexin contains two GTPase activating protein (GAP) domains, including one GTPase-binding domain and one PDZ domain (postsynaptic protein PSD-95/SAP90, the *Drosophila* septate junction protein Discs-large, and the tight junction protein ZO-1 domain) (B-type plexins only). In invertebrates, semaphorins 1 and 2 signal through Plexin A and Plexin B. In vertebrates, semaphorins 3, 5, and 6 signal via Plexin A, while semaphorin 3 requires a combination of neuropilins (Nrp1 or Nrp2) for signal transduction. Neuropilin is a transmembrane receptor composed of two complement-like (CUB) domains, two FV/FVIII clotting factor-like domains, one meprin-like MAM domain, and a short cytoplasmic tail.

Recently, emerging evidence suggests the semaphorin family in atherosclerosis plays a role. Semaphorins are a large family of transmembrane (including GPI-anchored) or secreted proteins that were originally identified as indispensable regulators of neuron-axonal guidance (Kolodkin et al., 1993). Their molecular structure consists of a highly conserved sema domain, a plexin–semaphorin–integrin (PSI) domain, and distinct protein domains that further define semaphorins, including immunoglobulin-like (Ig), thrombospondin, and basic C-terminal domains (Zhou et al., 2008). More than 20 semaphorin family members have been identified that fall into eight classes (Semaphorin Nomenclature Committee, 1999; Wannemacher et al., 2011). Classes 1 and 2 belong to invertebrates, and classes 3 through 7 belong to vertebrates while class 8 is viral-encoded. In addition, classes 1 and 4–7 are membrane-associated, whereas those in classes 2, 3, and 8 are in a secreted form (Capparuccia and Tamagnone, 2009; **Figure 1**). Two groups of proteins, including plexins and neuropilins (Npns or Nrps), have been identified as the primary semaphorin receptors (Tamagnone et al., 1999). It is worthy to note that nine plexins that also contain the extracellular sema domains were found in vertebrates and fall into classes A–D (Wannemacher et al., 2011). Several recent studies on semaphorin structure confirmed that each sema domain of a semaphorin homodimer binds to a plexin-sema domain to promote plexin dimerization for signal transduction (Janssen et al., 2010; Nogi et al., 2010; Wannemacher et al., 2011; **Figure 1**). Plexins are associated with different co-receptors in distinct tissues to allow semaphorins to exert pleiotropic functions (Alto and Terman, 2017). Although semaphorins were originally identified as axon guidance molecules required for axon pointing to proper targets (Kolodkin et al., 1993), cumulative findings indicate that they are indispensable in diverse physiological processes (Zhou et al., 2008), including cardiomyogenesis (Toyofuku and Kikutani, 2007; Toyofuku et al., 2008), tumor neovascularization, metastasis (Giordano et al., 2002; Neufeld and Kessler, 2008; Capparuccia and Tamagnone, 2009), osteoclastogenesis (Takegahara et al., 2006), angiogenesis (Serini et al., 2003; Gu et al., 2005; Toyofuku et al., 2007), and immunomodulation (Kikutani and Kumanogoh, 2003; Suzuki et al., 2008; Nishide and Kumanogoh, 2018). In this review, we focus on the role of semaphorins and their receptors in the setting of atherosclerosis development. Attention will be placed on several semaphorin family members for their role in semaphorin-receptor signaling during atherogenesis.

## Sema3A

There are seven members of secreted class 3 semaphorins, named Sema3A through Sema3G, which were found to express in the nervous system (Hashimoto et al., 2004; Hou et al., 2008), immune system (Ji et al., 2009), lung (Martin-Satue and Blanco, 1999), osteogenesis (Ryynanen et al., 2017), and cancer cells (Herman and Meadows, 2007; Karayan-Tapon et al., 2008). They function through their receptors, the neuropilins and plexins, forming complexes in which neuropilins serve as the ligand-binding moiety and plexins act as the signal transduction component (Sharma et al., 2012b). The extensive expression pattern of class 3 semaphorins suggests their other functions beyond the nervous system. Sema3A, first designated as collapsin-1, was found to regulate stimulation-induced growth cone slump (Luo et al., 1993) and repulsion of neuron axon growth in the nervous system (Kolodkin et al., 1997; Tamagnone and Comoglio, 2000; Dent et al., 2004). Nevertheless, emerging evidence indicates that Sema3A branches out beyond the nervous system, especially in the regulation of the immune system. Sema3A suppresses B- and T-cell proliferation and activation by inhibiting actin cytoskeleton reorganization and alleviating generation of proinflammatory cytokines (Lepelletier et al., 2006; Vadasz and Toubi, 2014). In addition, it induces apoptosis of endothelial cells (Guttmann-Raviv et al., 2007) and monocyte-derived macrophages through its receptors containing neuropilin and PlexinA (Moretti et al., 2008; Vadasz et al., 2013). Furthermore, Sema3A functions as an inhibitor of integrin function (Serini et al., 2003) and tube formation in endothelial cells (Guttmann-Raviv et al., 2007).

**FIGURE 2:**
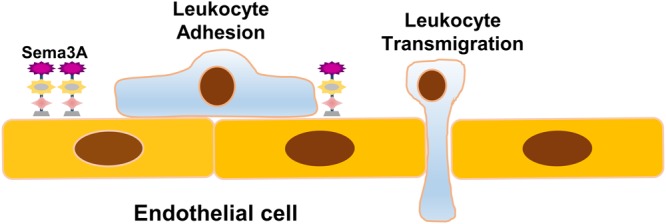
Schematic representation of the role of Sema3A in atherosclerosis. Sema3A functions as a natural barrier to prevent monocyte migration into the arterial intima under basal conditions. Sema3A is abundantly expressed by endothelial cells in the atheroresistent greater curvature while little is expressed in the lesser aortic curvature. The downregulation of Sema3A in the d-flow-injured endothelial cells leads to inflammatory leukocyte trafficking into the intima, an initial essential step in the process of atherogenesis, and accelerates inflammation and atherosclerosis development.

Recently, van Gils et al. (2013) revealed that endothelial Sema3A modulates leukocyte rolling, adhesion, and transmigration into the subendothelial wall during atherogenesis. They examined Sema3A expression in the inner and outer curvatures of the aortic arch of *low-density-lipoprotein receptor^-/-^* (*LDLR^-/-^*) mice following a western diet for 2 weeks and showed that Sema3A is abundantly expressed by endothelial cells in the antiatherogenic greater curvature, but little was detected in the proatherogenic lesser curvature. In *in vitro* analysis, Sema3A expression was obviously downregulated in human coronary artery endothelial cells (HCAECs) exposed to oscillatory (atheroprone) flow, proatherogenic factors, and proinflammatory cytokines. Sema3A acts as a barrier to prevent monocyte migration into the arterial intima as leukocyte rolling and adhesion to the endothelium was increased when Sema3A was inhibited using blocking peptides or blocking its receptor neuropilin-1. These data imply that Sema3A functions as a negative regulator of monocyte invasion, a priming step in the process of atherogenesis, alleviating inflammation progression and atherosclerosis development (**Figure 2**). In addition, endothelial Sema3A was reported to bind platelets and suppress platelet activation by inhibiting αIIbβ3 integrin-dependent spreading and granule releasing (Kashiwagi et al., 2005). Whether its antiplatelet effect contributes to atherosclerosis development is not clear.

## Sema3E

Semaphorin 3E (Sema3E), originally defined as M-SemaH, was identified in tumor cells and involved in embryonic development (Christensen et al., 1998). In the nervous system, Sema3E functions as a negative regulator of retinal ganglion cell axon growth and induces the collapse of retinal ganglion cell (RGC) axons via cGMP signaling (Steinbach et al., 2002). Further studies showed that the functions of Sema3E are associated with axon regeneration after injury (Sharma et al., 2012a; Bribian et al., 2014) and vascular development and remodeling (Gu et al., 2005; Kim et al., 2011; Oh and Gu, 2013). Sema3E suppresses endothelial cell motility and tube formation through the inhibition of VEGF-mediated Akt phosphorylation via PlexinD1 (Moriya et al., 2010; Aghajanian et al., 2014). Sema3E-PlexinD1 signaling in endothelial cells relies on the GTPase-activating protein (GAP) activity to modulate cytoskeleton reorganization and cellular migration and adhesion (Sakurai et al., 2010).

**FIGURE 3:**
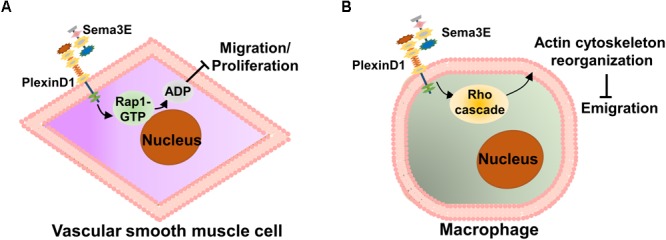
Schematic representation of the role of Sema3E in atherosclerosis. **(A)** In the early stage of atherosclerosis, Sema3E and its receptor PlexinD1 are remarkably decreased in atherosclerotic plaques. Sema3E inhibits VSMC migration and proliferation by inactivating Rap1-AKT signaling pathways via binding to PlexinD1 in the process of neointimal formation. **(B)** Sema3E and its receptor PlexinD1 are remarkably increased in monocyte-macrophages of advanced atherosclerotic plaques. Sema3E inhibits the directional emigration of macrophages by disrupting the Rho GTPase signaling cascade and actin cytoskeleton reorganization via binding to PlexinD1, which promotes monocyte-macrophage retention, exacerbating atherosclerosis.

Accumulating evidence suggests that the migration and proliferation of vascular smooth muscle cells (VSMCs) are crucial processes in neointimal formation, while neointimal hyperplasia is a pivotal pathophysiological process that contributes to atherosclerosis (Dzau et al., 2002; Owens et al., 2004; Gomez and Owens, 2012). To examine the role of Sema3E in VSMC migration and proliferation during neointimal formation, Wu et al. (2017) found that abundant Sema3E was expressed in alpha-smooth muscle actin (α-SMA)-positive VSMCs of mouse and human arteries under normal condition, while its expression in VSMCs of atherosclerotic plaques was markedly deceased as compared with the normal aortic arteries. Moreover, they found that Sema3E inhibited VSMC migration and neointimal formation in a dose-dependent manner *in vitro*. Mechanistically, Sema3E inactivated Rap1-Akt signaling pathways to suppress VSMC migration and proliferation via the PlexinD1 receptor. Therefore, Sema3E plays a negative regulatory role in the process of neointimal hyperplasia (**Figure 3A**).

In the later stage of atherosclerosis, as part of the resolution phase of acute inflammation, normally activated macrophages in the lesion emigrate from the site of local inflammation to the draining lymphatic vessels (Bellingan et al., 1996). However, unlike other inflammatory conditions, atherosclerotic cholesterol-laden macrophages (foam cells) persist in the arterial wall and the ability of emigration appears to be impaired (Llodrsa et al., 2004; Trogan et al., 2006). To date, the mechanisms by which these cells are retained in plaques remain poorly understood. Wanschel et al. (2013) investigated the dynamics of Sema3E expression in atherosclerosis using a model of atherosclerosis regression and identified Sema3E in atherosclerotic lesions, which are co-localized with infiltrated macrophages. Functionally, they also showed that Sema3E contributes to monocyte-macrophage invasion and accumulation in plaques. They further demonstrated that the decreased Sema3E mRNA level was accompanied by a downregulation of plaque macrophage accumulation and an increase in the proportion of M2 subtype macrophages. Besides, they showed that Sema3E regulates monocyte-macrophage retention and that its expression reduces along with plaque regression (Wanschel et al., 2013). Using bone marrow-derived macrophages (BMDM) *in vitro*, Sema3E expression was further investigated in macrophage polarization, which showed that Sema3E was highly upregulated in proinflammatory M1 subtype macrophages and that Sema3E inhibits the directional emigration of macrophages by disrupting the Rho GTPase signaling cascade, actin cytoskeleton reorganization, and polarization via binding to PlexinD1 (Wanschel et al., 2013). Thus, Sema3E appears to function as an inducer of monocyte-macrophage infiltration and an inhibitor of macrophage emigration and thus may accelerate atherosclerosis by promoting monocyte-macrophage retention and long-term inflammation (**Figure 3B**).

## Sema4D

Semaphorin 4D (Sema4D/CD100) is a 150-kD transmembrane protein that belongs to the semaphorin class 4. It was first perceived as an “immune system semaphorin” (Wang et al., 2001) and indispensably takes part in the immune responses (Kumanogoh and Kikutani, 2003). Two types of receptors with distinct binding affinities have been identified for Sema4D. PlexinB1 is a receptor that has been shown to have a relatively high affinity for Sema4D (Maestrini et al., 1996; Tamagnone et al., 1999), while CD72, expressed at prominent levels in lymphocytes, is a low-affinity receptor for Sema4D. The interaction between Sema4D and its receptor CD72 is critical for immune regulation (Chapoval et al., 2017), including lymphocyte activation, production of antibody, and autoimmune diseases (Wang et al., 2001).

**FIGURE 4:**
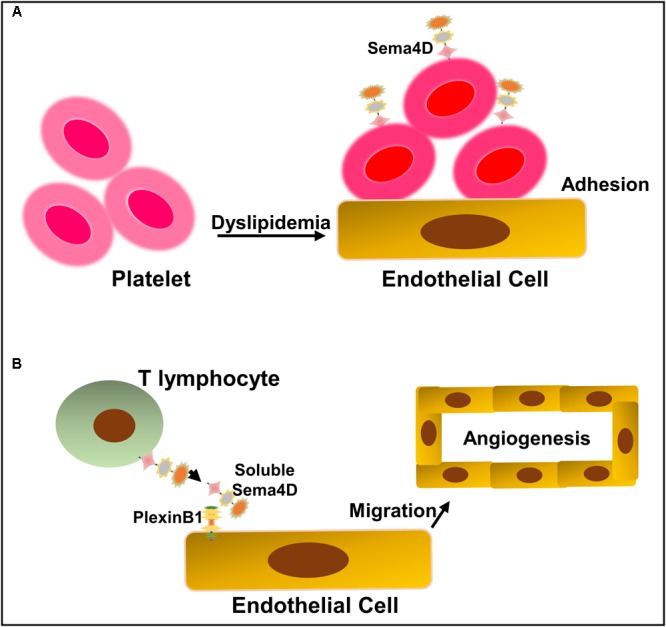
Schematic representation of Sema4D and its receptor PlexinB1 in the development of atherogenesis. **(A)** Dyslipidemia upregulates platelet sensitivity, making them more likely to become activated in response to vascular injury. Sema4D enhances platelet hyperactivity, which may lead to platelet adhesion and transmigration into the intima, thus accelerating the development of atherosclerosis. **(B)** Sema4D expresses on the surface of invasive lymphocytes in atherosclerotic lesions and interacts with PlexinB1 on the endothelial cells, resulting in endothelial cell activation, macrophage infiltration, and intraplaque angiogenesis.

Sema4D is the first semaphorin family member that was shown to participate in the development of atherosclerosis (Zhu et al., 2009). This finding was largely based on the previous observations that Sema4D promotes platelet activation *in vitro* and accelerates thrombus formation and growth (Zhu et al., 2007; Wannemacher et al., 2010). Using Sema4D-deficient *LDLR^-/-^* mice, Zhu et al. (2007, 2009) investigated whether eliminating Sema4D-dependent events could provide a means of platelet hyperactivity reduction in the setting of dyslipidemia. They found that the loss of Sema4D expression ameliorates the effects of dyslipidemia on the platelet function *in vivo* and *ex vivo*, and reduces atherosclerotic lesions in hyperlipidemic mice on a high-fat, high-cholesterol diet for 3 or 6 months (Zhu et al., 2009) (**Figure 4A**). Platelet is not the unique source of Sema4D expression as T cells, B cells, and monocytes/microphages have been reported to express Sema4D (Bougeret et al., 1992; Hall et al., 1996). Luque et al. (2013, 2015) showed that Sema4D is expressed in plaque macrophages and foam cells and mediates monocyte-endothelial cell adhesion by interacting with its receptors, PlexinB2 and PlexinB1, implying that non-platelet Sema4D may participate in atherogenesis as well. However, Zhu et al. (2007, 2009) did not observe the reduction in the infiltration of Sema4D-expressing cells, including T cells, B cells, and monocytes/macrophages in atherosclerotic lesions when investigating whether the reduction of hyperlipidemia is by the global absence of Sema4D. Whether the role of Sema4D in the platelet function is directly related to its ability to enhance atherogenesis needs to be examined by a megakaryocyte-selective deletion of Sema4D.

The neovasculature originates from the adventitia to nourish blood vessels and the thickened atherosclerotic intima (Kumamoto et al., 1995; Moreno et al., 2006). Neovascularization in atherosclerotic plaques is thought to exacerbate atherosclerosis by fueling plaques with metalloproteases from blood, resulting in plaque rupture and thrombosis (Jonsson-Rylander et al., 2005). Therefore, neovascularization is considered to be one of the major causes of atherosclerotic plaque growth and destabilization (Barger et al., 1984). Subsequent to the role of Sema4D in enhancing platelet hyperreactivity in atherosclerosis, Yukawa et al. (2010) reported that it accelerates atherosclerosis by facilitating intimal neovascularization and monocyte-macrophage infiltration in apolipoprotein E (ApoE)-deficient mice. Furthermore, they found that Sema4D was expressed in the invasive lymphocytes of atherosclerotic lesions and that the degree of plaque neovascularization in *Sema4D^-/-^ ApoE^-/-^* mice was significantly reduced. They concluded that Sema4D plays an integral role in the development of atherosclerosis by promoting neovascularization of the intima, facilitating macrophage infiltration in atherosclerotic plaques.

The mechanism for the role of Sema4D in angiogenesis has been well studied. It is not likely to be mediated by upregulating VEGF or angiogenin (Conrotto et al., 2005). A study on tumor-induced angiogenesis showed that Sema4D may function as a direct inducer of endothelial cell migration to promote neovascularization instead of promoting endothelial cell growth like VEGF. It has been found that angiogenesis has a spatial preference for atherosclerosis (Moulton, 2001; Moulton et al., 2003). Therefore, unlike VEGF, Sema4D may regulate neovascularization in atherosclerotic plaques by modulating local endothelial cell migration. In-depth studies showed that Sema4D exerts proangiogenic efficacy by binding to PlexinB1 that is expressed on the surface of endothelial cells. Instead of activating the Rho kinase pathway, the binding of Sema4D to PlexinBl activates the Met receptor for signal transduction (Basile et al., 2004; Conrotto et al., 2005). Interestingly, R-Ras, whose activity is downregulated during the signal transduction of Sema4D to PlexinBl, also functions to inhibit intimal growth and tumor angiogenesis (Komatsu and Ruoslahti, 2005). Similarly, studies conducted by Yukawa et al. (2010) indicated that cleaved Sema4D from infiltrated T lymphocytes may promote atherosclerosis by inducing the migration of regional endothelial cells to form new blood vessels, causing macrophage infiltration and atherosclerosis development (**Figure 4B**).

## Sema7A

Semaphorin 7A (Sema7A) is the only membrane-associated glycosylphosphatidylinositol (GPI)-anchored semaphorin (Xu et al., 1998) with an N-terminal seven-bladed β-propeller sema domain, a plexin-semaphorin-integrin domain, an immunoglobulin-like domain, and a C-terminal GPI-anchoring domain (Lange et al., 1998; Liu et al., 2010). The sema domain contains an RGD motif commonly found in integrin-binding proteins (Pasterkamp et al., 2003). The expression of Sema7A and its known membrane receptors, integrin β1 and PlexinC1, were found in neurons (Pasterkamp et al., 2007), endothelial cells (Morote-Garcia et al., 2012; Hu et al., 2018), platelets (Jaimes et al., 2012), monocytes (Holmes et al., 2002), T cells (Czopik et al., 2006; Suzuki et al., 2007), dendritic cells (DCs) (Scott et al., 2008), lung fibroblasts (Esnault et al., 2017), and cancer cells (Garcia-Areas et al., 2013, 2014, 2017). The expression and binding properties of Sema7A suggest that it could play an important role in both the adult nervous system and immune function modulation (Xu et al., 1998). It has been reported that Sema7A complexes with the semaphorin-binding module of PlexinC1. Two PlexinC1 molecules are symmetrically bridged by Sema7A dimers, in which the Sema7A and PlexinC1 β propellers interact (Liu et al., 2010).

The first evidence for Sema7A in the nervous system was provided by the work on axon outgrowth in the olfactory system. Sema7A promotes the outgrowth of olfactory bulb axons and is required for the development of the lateral olfactory tract that carries olfactory bulb axons (Pasterkamp et al., 2003). Increasing evidence suggests that Sema7A plays important roles beyond the nervous system. Sema7A binding to PlexinC1 mediates tumor growth (Scott et al., 2009), while Sema7A binding to integrin β1 initiates T-lymphocyte-mediated inflammatory-immune response (Suzuki et al., 2007). More recently, Sema7A is implied in endothelial homeostasis. When exposed to hypoxia, Sema7A expression is significantly upregulated in vascular endothelial cells and promotes neutrophil adhesion and transmigration to the subendothelial layer (Morote-Garcia et al., 2012). Besides, Sema7A was reported to stimulate monocyte chemotaxis and cytokine production. Suzuki et al. (2007) showed that Sema7A, which is highly upregulated on activated T lymphocytes, irritates monocytes and macrophages to produce inflammatory cytokines via integrin α1β1.

**FIGURE 5:**
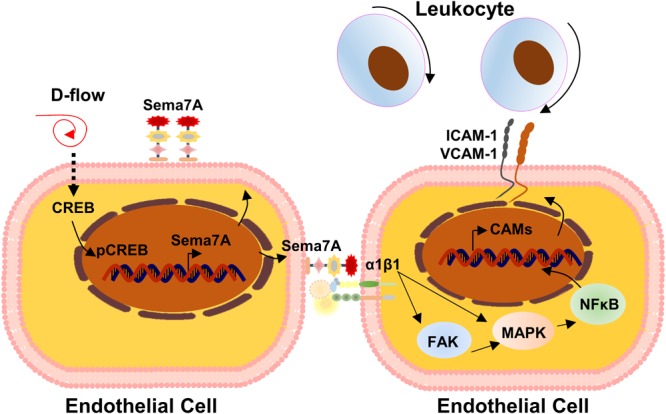
Mechanistic diagram of Sema7A in atherosclerosis. Endothelial Sema7A is upregulated in response to d-flow in the lesser curvature of mouse aortic arch, which is potentially regulated by the inhibition of cAMP response element-binding protein (CREB) signaling. Sema7A promotes the expression of adhesion molecule ICAM-1/VCAM-1 via NF-κB activation and leukocyte recruitment through integrin β1, accelerating atherosclerosis.

Given the wide expression of Sema7A and its receptors in blood cells (Garcia-Areas et al., 2013) and its well-known function in immunity and inflammation, Hu et al. (2018) recently hypothesized that Sema7A may be an inflammatory responsive protein in the endothelial cells and examined the role of Sema7A in atherogenesis using *ApoE^-/-^* mice. They showed that Sema7A deficiency attenuates plaque formation in *ApoE^-/-^* mice on a high-fat diet, with reduced accumulation of macrophages in the plaques (Hu et al., 2018). Interestingly, the reduction in the plaque size was primarily in the aortic arch exposed to d-flow. Further studies showed that Sema7A is upregulated in the lesser curvature of mouse aortic arch endothelium, carotid artery exposed to d-flow in mice subjected to partial carotid artery ligation (PCL), and human umbilical venous endothelial cells (HUVECs) under oscillatory shear stress, potentially associated with the inhibition of cAMP response element-binding protein (CREB) signaling. Functionally, Sema7A deficiency remarkably reduced leukocyte rolling and adhesion on TNF-α-primed endothelium and leukocyte–endothelium interaction. Mechanistically, Sema7A overexpression enhances intercellular cell adhesion molecule-1 (ICAM-1) and vascular cell adhesion molecule-1 (VCAM-1) expression and THP-1 adhesion to HUVECs in a β1 integrin-dependent manner that involves its downstream FAK/MAPK/NF-κB signaling pathway. They concluded that Sema7A upregulation by d-flow mediates endothelial dysfunction and atherosclerosis in a β1 integrin-dependent manner (**Figure 5**).

## Miscellaneous

In addition to Sema3A, Sema3E, Sema4D, and Sema7A that have been extensively described for their role in the development of atherosclerosis, other members of the semaphorin family are potentially involved in atherogenesis due to their role in endothelial activation and dysfunction, neovascularization, and immune response. Semaphorin 6A (Sema6A), a transmembrane semaphorin protein expressed in endothelial cells, sustains the homeostasis of endothelial cells by regulating the vascular endothelial growth factor receptor 2 (VEGFR2) expression and signaling (Segarra et al., 2012). Segarra et al. (2012) identified Sema6A as a crucial regulator of vascular development and potential therapeutic target for ocular pathologies by contributing to the formation and remodeling of hyaloid vessels, indicating that Sema6A may be involved in angiogenesis and vessel remodeling that play indispensable roles in advanced plaque development.

Atherosclerosis is the consequence of a chronic inflammatory response in the arterial wall and the activation of the innate immune system has been recognized to be fundamentally involved in atherogenesis (Chavez-Sanchez et al., 2014). More recently, evidence has been provided that adaptive immunity is also involved in the initiation and progression of atherosclerosis (Luchtefeld et al., 2010). In particular, Sema4A has been investigated extensively. Expressed on dendritic cells and B-lymphocytes, Sema4A increases the activity of T lymphocytes (Suzuki and Kumanogoh, 2006), including cell activation and differentiation, and regulates the generation of T lymphocytes both *in vitro* and *in vivo* (Kumanogoh et al., 2002). However, there is no report on whether Sema4A participates in atherosclerosis. Like Sema4A, Sema4D, Sema3A, and Sema7A, reported to participate in atherosclerosis, are also widely investigated in immune cells. Sema4D has been shown to be critical for B-lymphocyte (Kuklina et al., 2017) and dendritic cell activation (Wang et al., 2001; Suzuki et al., 2003). Sema3A is highly expressed on many immune cells and responsible for T-cell and dendritic cell activation, inhibiting T-cell proliferation and proinflammatory cytokines production (Lepelletier et al., 2006; Vadasz and Toubi, 2014). Suzuki et al. (2007) reported that Sema7A, abundantly expressed on activated T lymphocytes, may potently stimulate monocyte-macrophages at the immunological synapse where Sema7A protein accumulates at the contact site between T lymphocytes and macrophages. Therefore, semaphorins in the immune system may provide a link between immune response and atherosclerosis. How such mechanisms of semaphorins would be started up in response to inflammatory injury in the context of atherosclerosis remains to be investigated.

Semaphorins, netrins, slits, and ephrins make up the four families of neuronal guidance cues that regulate neuronal growth and migration via multiple signaling pathways. Growing evidence suggests that members from other families participate in the regulation of atherosclerosis. Similar to Sema3A, netrin-1 was found to be involved in the initiation of atherosclerosis. Netrin-1 was detected by immunostaining in endothelial cells of the greater curvature but was significantly downregulated in endothelial cells at the lesser curvature of the aortic arch. *In vitro*, netrin-1 suppresses leukocyte recruitment by inhibiting the production of proatherogenic chemokines monocyte chemotactic protein-1 (MCP-1) and fractalkine. A consistent result was observed in the adhesion of monocytes to endothelial cells, suggesting that netrin-1 functions as a negative regulator of atherosclerosis (van Gils et al., 2013). Besides, recent studies showed that Slit2 inhibits monocyte adhesion on activated human endothelial cells and chemotaxis of monocytes to chemokines stromal cell-derived factor-1 (SDF-1) and MCP-1. In addition, the inhibition of monocyte recruitment by supplementing Slit2 was found to delay atheroprogression in mice (Mukovozov et al., 2015). On the other hand, ephrinB2 was demonstrated to be upregulated in endothelial cells by proatherogenic stimuli and remarkably promote monocyte recruitment (Poitz et al., 2015).

## Conclusion

**FIGURE 6:**
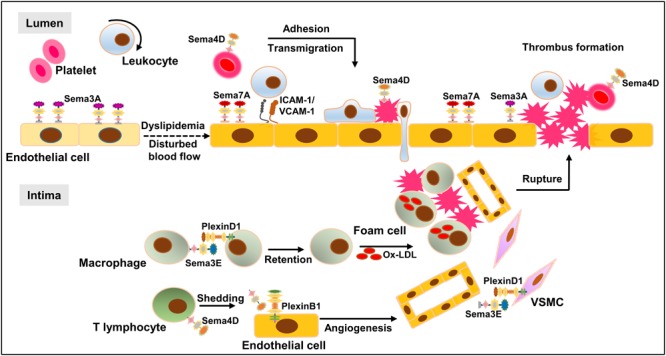
Network regulation of semaphorins in atherosclerosis. When exposed to oxLDL or injured by disturbed blood flow, endothelial cells undergo major phenotypic changes, e.g., upregulation of inflammatory cytokines, leading to leukocyte adhesion and infiltration into the intima wall. Accumulating immune cells including macrophages and T cells fuel the immune response, leading to the transition of a fatty streak to a complex atherosclerotic plaque. Plaque rupture and thrombus formation may lead to fatal cardio-cerebrovascular events. D-flow upregulates endothelial Sema7A and downregulates endothelial Sema3A and VSMC-Sema3E, leading to the infiltration of inflammatory leukocytes and VSMCs. Following cell infiltration and plaque formation, intraplaque Sema4D, shedding from activated T lymphocytes, interacts with PlexinB1 on endothelial cells, resulting in endothelial cell migration and intraplaque angiogenesis. In the advanced plaque, Sema3E interacts with PlexinD1 on macrophages to regulate macrophage emigration. Upon plaque rupture, platelet Sema4D binds leukocytes as well as injured endothelial cells to support thrombus formation, exacerbating the pathological process.

Growing evidence indicates that semaphorins have distinct biological activities, which are not only limited to the nervous system but also branching into other pathophysiological processes, including inflammation, immune response, angiogenesis, and especially atherogenesis, of which several mechanisms work in concert to modulate the initiation, progression, and regression of atherosclerosis. When exposed to disturbed blood flow or injured by inflammatory factors, endothelial cells upregulate Sema7A and downregulate Sema3A while VSMCs reduce Sema3E expression. The upregulation of Sema7A and downregulation of Sema3A and Sema3E function as proatherogenic factors to induce cell adhesion and transmigration via several mechanotransduction pathways. Meanwhile, inflammatory stimulation may induce platelet Sema4D activation, enhancing leukocyte adhesion and transmigration into the subendothelium to accelerate the development of atherosclerosis. In the plaques, Sema4D may shed from activated T lymphocytes and interact with PlexinB1 on endothelial cells, causing endothelial cell activation, leukocyte migration, and intraplaque angiogenesis. Subsequently, Sema3E interacts with PlexinD1 on macrophages to impair their emigration from advanced plaques, thereby promoting macrophage retention and chronic inflammation. Upon plaque rupture, platelet Sema4D binds to leukocytes and injured endothelial cells to exacerbate thrombosis and inflammation (**Figure 6**). Future investigation using the state-of-the-art technologies on the role of additional semaphorin family members in atherogenesis, their potential network regulation in atherosclerosis and their association with other proatherosclerotic or antiatherosclerotic factors will not only shed light on the full picture of the functional role of semaphorins in atherogenesis but also identify potential therapeutic targets and pharmacological interventions for atherosclerosis.

## Author Contributions

SH selected topics and wrote the review. LZ reviewed the manuscript and modified the content. All authors critically read and commented on the manuscript.

## Conflict of Interest Statement

The authors declare that the research was conducted in the absence of any commercial or financial relationships that could be construed as a potential conflict of interest.
